# *Rosmarinus officinalis* and Methylphenidate Exposure Improves Cognition and Depression and Regulates Anxiety-Like Behavior in AlCl_3_-Induced Mouse Model of Alzheimer’s Disease

**DOI:** 10.3389/fphar.2022.943163

**Published:** 2022-08-12

**Authors:** Nishat Malik, Sanila Amber, Saadia Zahid

**Affiliations:** Neurobiology Research Laboratory, Department of Healthcare Biotechnology, Atta Ur Rahman School of Applied Biosciences (ASAB), National University of Sciences and Technology (NUST), Islamabad, Pakistan

**Keywords:** Rosmarinus officinalis, methylphenidate (MPH), anxiety, depression, cognition

## Abstract

Alzheimer’s disease (AD) is a neurological illness that causes severe cognitive impairment. AD patients also experience at least one of the neuropsychiatric symptoms including apathy, depression, and anxiety during the course of their life. Acetylcholine esterase inhibitors are the available treatment options to alleviate cognitive deficits, whereas methylphenidate (MPH), a psychostimulant, is considered for the treatment of apathy in AD patients. *Rosmarinus officinalis*, a perennial herb, has been potentially known to have antioxidant and anti-inflammatory properties. The present study investigated the potential effects of MPH and *R. officinalis* in comparison with the standard drug, Donepezil, on cognition, anxiety, and depression in the AlCl_3_-induced mouse model of AD. The animals were divided into eight groups (n = 8, each). The results revealed that the MPH- and *R. officinalis*-treated groups significantly improved memory impairment, whereas *R. officinalis* substantially reduced depression and anxiety as compared with other treatment groups. MPH treatment induced an antidepressant effect and increased anxiety-like behavior. Moreover, the AlCl_3_ exposure led to the formation of amyloid beta (Aβ) plaques in mice hippocampus; however, none of the tested drugs caused a significant reduction in amyloid burden at the selected doses. The present study suggested the potential of *R. officinalis* to improve memory as well as neuropsychiatric symptoms in AD. Although *R. officinalis* improved cognitive abilities, it did not reduce the amyloid plaque burden, which indicates that the memory-enhancing effects of *R. officinalis* are due to some alternate mechanism that needs to be explored further.

## Introduction

Alzheimer’s disease (AD) is a neurological illness that causes neuronal loss and cognitive impairment. The major pathological characteristic of the disease is the successive accumulation of amyloid beta (Aβ) plaques and neurofibrillary tangles (Scheltens et al., 2021). AD has public health burden because of not just cognitive symptoms but also noncognitive neuropsychiatric symptoms including apathy, agitation, depression, aggression, and anxiety. During the course of the disease, most of the AD patients will have at least one of these symptoms (Heilman and Nadeau, 2022). Apathy affects 70% of people with AD, whereas depression and anxiety are also evident in AD patients (Teixeira et al., 2021). The occurrence of depression and anxiety in AD patients has a significant ramification on the patient’s quality of life, wellness of the caregivers, chances of hospitalization, and mortality rate (Botto et al., 2022).

A number of pharmaceutical treatments for treating apathy in AD have also been investigated. Various randomized clinical trials of cholinesterase inhibitors have shown minor improvements in apathy-associated symptoms (Sepehry et al., 2017). Furthermore, antidepressants are also unable to improve the apathy-related symptoms in AD patients, and some studies reported the negative consequences of these medications (Magierski et al., 2020).

Methylphenidate (MPH) is a potent psychostimulant that has been utilized to enhance cognition and promote wakefulness for a range of disorders (Wenthur 2016). It has become the standard treatment for attention deficit hyperactivity disorder with time, because of its ability to decrease impulsivity and improve cognition and executive control (Shellenberg et al., 2020).

Based on clinical anecdotal reports, MPH is being considered for the treatment of apathy in AD, and preliminary trials have shown promising outcomes (Padala et al., 2018; Mintzer et al., 2021).

Plant-originated natural compounds having pharmacological properties have appeared as a promising treatment alternative for AD in recent years (Uddin et al., 2020; Akter et al., 2021; Fernandes et al., 2022). These natural compounds or phytochemicals are largely categorized into alkaloids, terpenoids, and polyphenols, where the terpenoids and polyphenols are the major groups of plant’s secondary metabolites, targeting several signaling pathways in the biological system (Zhu et al., 2018). The pharmacological properties of these compounds are due to their distinctive structures that enable them to interact with different key enzymes, receptors, antioxidant systems, and signaling cascades including transcription factors as well as cytokines (Safe et al., 2021; Santos et al., 2021; Kamran et al., 2022). For instance, the antioxidant activity of flavonoids, a subgroup of polyphenols, is correlated with the number of phenolic groups (Glevitzky et al., 2019), whereas anti-inflammatory and antidepressant effects are also associated with polyphenols (Hussain et al., 2016; Jiang et al., 2019). Moreover, terpenoids are considered for exhibiting anticholinesterase activity and are a promising source for future AD treatment (Lai Shi Min et al., 2022).

*Rosmarinus officinalis* (*R. officinalis*), having the common name rosemary, is a member of the Lamiaceae family and is rich in phenolic and terpenoid compounds (Andrade et al., 2018). It is potentially known to have antioxidant, anti-inflammatory, and antidepressant properties (Guo et al., 2018; Dabaghzadeh et al., 2022).

At present, there is no effective medicine that can cure or stop the deterioration of neurons and manage multiple symptoms of AD at a time, although many new drugs are under clinical trials proposed as neuroprotective treatment (Huang et al., 2020). Cholinesterase inhibitors including Donepezil are the only available treatments for managing cognitive symptoms in AD (Haake et al., 2020). However, they are associated with several adverse side effects (Zhang et al., 2022). A recent study demonstrated the potential anticholinesterase activity of *R. officinalis* to combat cognitive decline disorders (Kamli et al., 2022). By contrast, our earlier findings also highlighted the potential of *R. officinalis* to enhance memory and affect synaptic regulation in the Aβ_1–42_-induced AD mouse model (Mirza et al., 2021). Likewise, another study by our group showed the therapeutic potential of *R. officinalis* and MPH to improve cognition and regulate inflammation, synaptic gene expression, and hippocampal neuronal density in mouse models of AlCl_3_-induced neurotoxicity (Khalid et al., 2020).

Based on the promising results of our previous work and the available literature, the current study focused on the potential effects of MPH and *R. officinalis* on anxiety, depression, and cognition through behavioral analysis in an AlCl_3_-induced mouse model of AD, to explore a better and comprehensive therapeutic regimen that can manage multiple symptoms including memory loss and psychiatric symptoms. The study highlights the potential effects of *R. officinalis* and MPH on psychological behaviors suggesting it as a better option to treat neuropsychiatric as well cognitive symptoms associated with AD. However, further studies are needed to explore the molecular mechanisms regulated by *R. officinalis* to help understand its mode of action.

## Materials and Methods

### Reagents and Drugs

Aluminum chloride hexahydrate (AlCl_3_·6H_2_O, Cat #: AL0770) was procured from Pharmpur^®^ Scharlau, Spain. Donepezil hydrochloride (Donecept) was from ATCO Laboratories, Pakistan, and methylphenidate hydrochloride (Ritalin^®^) was procured from Novartis specs, Pakistan. All chemicals were obtained from Merck, Germany, unless noted otherwise, and were of molecular biology grade.

### Animals

Male BALB/c mice (6–8 weeks old) were chosen for the study. Animals were bred and resided in the Laboratory Animal House of Atta-ur-Rahman School of Applied Biosciences (ASAB), National University of Science and Technology (NUST). Mice (n = 64) were housed in standard metal cages under standard conditions of constant temperature (25°C ± 2°C) and a regular light–dark cycle (12–12 h). All experimental protocols were conducted in accordance with the rulings of the Institute of Laboratory Animal Research, Division on Earth and Life Sciences, National Institute of Health, United States (Guide for the Care and Use of Laboratory Animal). Internal Review Board of ASAB, NUST approved this study protocol.

### *Rosmarinus officinalis* Extract Preparation

Commonly available dried leaves of *R. officinalis* were collected during the fall of 2020 from a vendor in the local spice market of Islamabad, Pakistan. Verification of the plant was conducted by an experienced botanist prior to the initiation of the procedure. The specimen was stored in Neurobiology Lab, ASAB, NUST, and also submitted at Pakistan Natural History Museum, Islamabad, Pakistan, with voucher no. 42570. *R. officinalis* extract was prepared according to the protocol described by Khalid et al. (2020). *R. officinalis* (500 g) leaves were ground to fine powder form and allowed to pass through 80 mesh sieves. It was followed by taking 10 g of fine powder in a thimble and loading the thimble into Soxhlet extractor with a distillation flask containing 100% ethanol as extraction solvent. The process was run for 24 h before filtrate was collected and concentrated using a rotary evaporator (R200 Rotavapor, Buchii) under the pressure of 68°C to attain a crude extract. The crude extract was incubated at 37°C to remove the remaining solvent. The extract was stored at 4°C until further use.

### Animal Treatment

**FIGURE 1:**
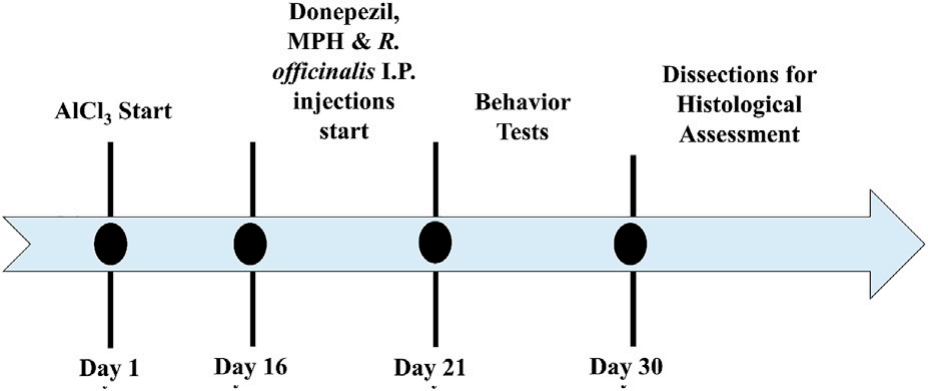
Timeline depicting a period for the induction of Alzheimer’s disease (AD), treatment with Donepezil, methylphenidate (MPH), and *R. officinalis* extract; behavior analysis; and decapitation of animals for histological assessment.

Animals were divided into eight groups, eight mice each. Animals of groups 1, 2, 3, and 4 were given distilled water and normal feed for 15 days. After 15 days, the animals of groups 2, 3, and 4 were administered with intraperitoneal (i.p.) injections (single injection volume of 100 µl) of 2 mg/kg (i.p.) Donepezil (Madani Neishaboori et al., 2021), 10 mg/kg (i.p.) MPH, and 100 mg/kg (i.p.) *R. officinalis* extract (Khalid et al., 2020) for 5 days. Aluminum chloride (AlCl_3_) (300 mg/kg) (Amber et al., 2018; Khalid et al., 2020) was given in drinking water with normal feed for 15 days to groups 5, 6, 7, and 8 (n = 32). After the development of AlCl_3_-induced AD models, the animals of groups 5, 6, 7, and 8 were switched to normal drinking water and those of groups 5, 6, and 7 were administered with intraperitoneal (i.p.) injections (single injection volume of 100 µl) of 2 mg/kg (i.p.) Donepezil, 10 mg/kg MPH, and 100 mg/kg *R officinalis* extract, respectively. Behavior tests were conducted in the next 10 days followed by dissection, collection of brain tissues, and histological assessment. The treatment timeline is depicted in Figure 1.

### Behavior Studies

#### Morris Water Maze Test

The procedure described by (Bromley-Brits et al., 2011) was used with small modification for the Morris water maze (MWM) test. A circular pool (120 cm × 60 cm), divided equally into four quadrants (east, west, north, and south), was filled with water (21°C ± 2°C). A transparent platform (13 cm × 32 cm) was placed 1 cm below the surface of the water in the north–west quadrant. Five acquisition trials were conducted five times a day for five consecutive days, and before each trial, a minimum of 10 min intertrial interval was kept for each mouse. In each trial, the mice were released in water at one of the four but different quadrant positions with their heads facing the tank. The cut-off time was identified as 90 s, and those who failed to locate the platform in 90 s were manually placed on the platform for 20 s. Those animals who located the platform before 90 s were allowed to sit there for 5 s. The escape latency for all the trials of 5 days was recorded, and the average was calculated. On day 6, the platform was removed and a probe test was conducted. In this test, the mice were allowed to swim in the pool for 90 s and the video was recorded. The animal’s reference memory was monitored by calculating the number of entries, time spent in the target quadrant, and number of crossings made by the subjects over the removed platform position.

#### Forced Swim Test

A forced swim test was conducted to analyze the depressive behavior of the animals. The protocol of the test was followed as described by (Murad et al., 2014) with few modifications. A transparent container with a diameter of 20 cm and a height of 30 cm was filled with water at 25°C ± 2°C. The depth of the water was adjusted according to the size of the mice, to prevent their hind limbs and tail from touching the bottom of the container. The animals were placed into the water, and their activity was recorded using a camera for 6 min. The water in the container was discarded and refilled for each mouse prior to testing. The time spent immobile, number of immobile episodes, and latency to immobility were calculated for each mouse.

#### Open Field Test

An open field test was conducted to assess the anxiety level and exploratory behavior of the animals. Protocol for the test was followed as described by (Farhat et al., 2017) with slight modifications. A square-shaped arena (40 × 40 × 40) was used, which was divided into center and periphery by drawing a boundary. Each mouse was placed in the center of a wooden box and was allowed to explore the box for 30 min. The behavior of the animals was monitored using a camera and was assessed by calculating the time spent in the center and periphery of the box.

#### Statistical Analysis

Data were analyzed using GraphPad Prism 8.0.2 by applying a one-way analysis of variance (ANOVA) followed by Bonferroni’s multiple comparison test. Two-way ANOVA was applied for analyzing escape latency in the MWM test. The data observed are shown as mean ± SEM having a 95% confidence interval, which is considered statistically significant when *p* < 0.05.

### Histological Examination

Heart perfusion was conducted to excise the whole brain according to the procedure described by Gage et al. (2012). Tissue sections (5 µm) of the hippocampus were deparaffinized with xylene, rehydrated, and washed with 70% isopropanol and double-distilled water (ddH_2_O) respectively. The Congo red stain (Cat #C6767, Merck Germany; working solution: 49.5 ml Congo red (Stock) and 0.5 ml 1% NaOH) was poured on the deparaffinized brain sections and retained for 20 min. ddH_2_O and alkaline alcohol were used to wash sections for 2 min. The sections were then counterstained by hematoxylin for 30 s and further washed with 70% isopropanol for 6 min and then with ddH_2_O. After air-drying (1 h), the slides were mounted by coverslips and later visualized using B-150, OPTIKA microscope (Italy) at 40×, 10×, and 4× resolution. The images were captured using Optika Vision Lite 2.1 image analysis software.

## Results

### MPH and *R. officinalis* Improved Cognition

**FIGURE 2:**
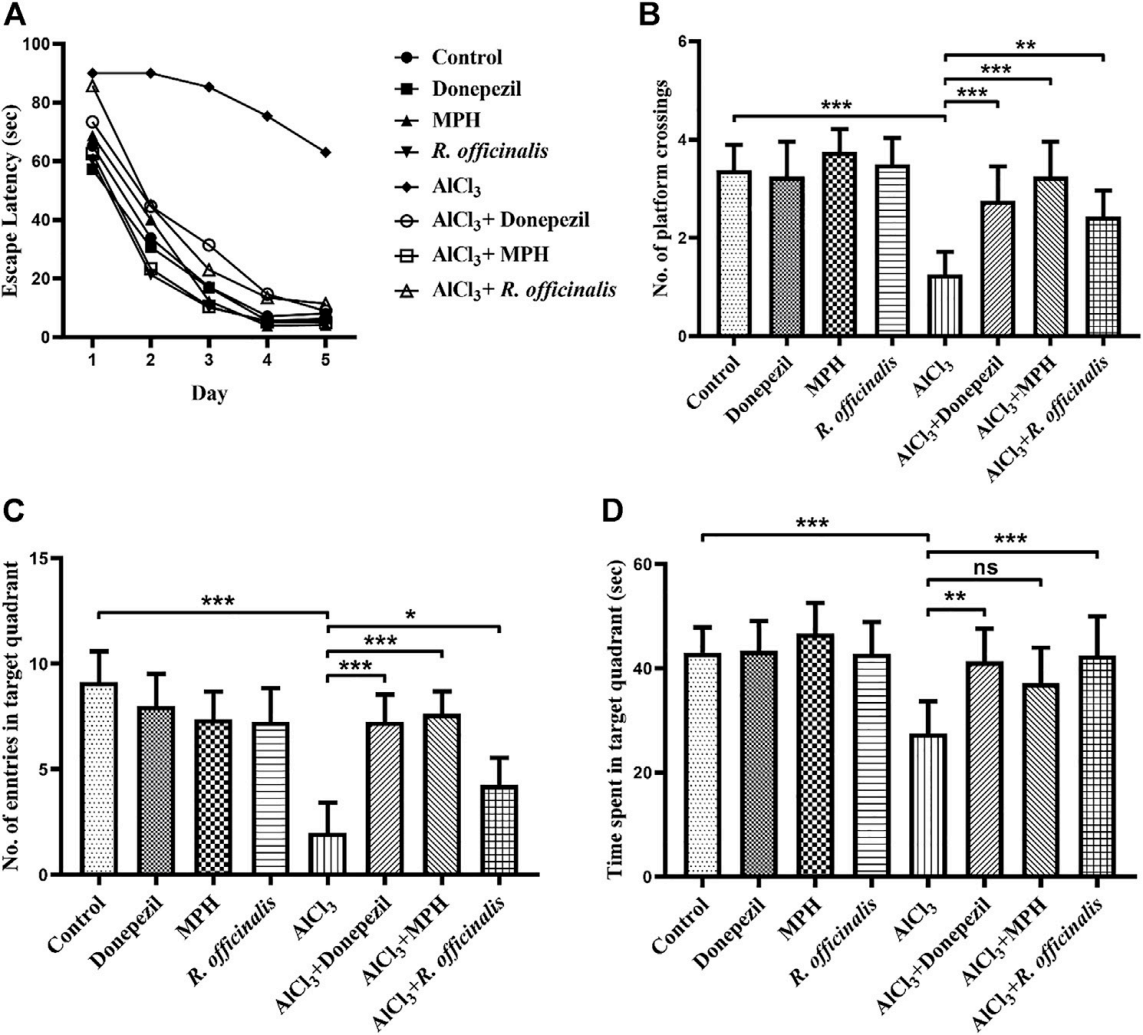
Effects of Donepezil, MPH, and *R. officinalis* on spatial memory in AlCl_3_-induced mice models using the Morris water maze test. **(A)** Graph demonstrates escape latency (sec) of the experimental groups, **(B)** number of platform crossings by the study groups, **(C)** number of entries in the target quadrant, and **(D)** time (sec) spent in the target quadrant during the probe trial. One-way ANOVA followed by the Bonferroni comparison test (mean ± SEM) was applied to analyze the data using GraphPad Prism. n = 8. ^***^ *p* < 0.001, ^**^ *p* < 0.01, ^*^ *p* < 0.05.

The MWM test was used to assess the outcomes of MPH and *R. officinalis* on spatial learning and memory in comparison with Donepezil. Average escape latency to find out the platform directly indicates the effects of the drugs on spatial memory. The AlCl_3_-treated group showed poor memory retention than the control group by demonstrating an escape latency of 63 s on day 5. A significant improvement (*p* < 0.01) in spatial memory was observed in the AlCl_3_ + *R. officinalis*-treated group. However, better (*p* < 0.05) restoration of spatial memory was seen in the AlCl_3_ + MPH- and AlCl_3_ + Donepezil-treated groups. Escape latency for 5 days is represented graphically in Figure 2A.

A probe trial was conducted for the assessment of reference memory and exploratory behavior of mice for the previously placed invisible platform in the target quadrant. The AlCl_3_-treated group reflected a significant decrease (*p* < 0.0001) in reference memory by making a smaller number of crossings over the platform position as compared with the control group. A number of platform crossings were significantly increased in the AlCl_3_ + *R. officinalis*-treated group as compared with the AlCl_3_-treated group; however, the AlCl_3_ + Donepezil- and AlCl_3_ + MPH-treated groups made a greater number of crossings than the AlCl_3_ + *R. officinalis*-treated group (Figure 2B). A similar trend was seen in the number of entries where the AlCl_3_-treated group showed the least number of entries in the target quadrant than the control and all the other experimental groups. Again, the AlCl_3_ + Donepezil- and AlCl3 + MPH-treated groups showed a greater number of entries than the AlCl_3_ + *R. officinalis-*treated group (Figure 2C). Likewise, a significant decrease (*p* < 0.001) in time spent in the target quadrant was observed in the AlCl_3_-treated group compared with the control group and a significant improvement was observed after treatment with *R.* officinalis and Donepezil in the AlCl_3_ + *R. officinalis-* and AlCl_3_ + Donepezil-treated groups (Figure 2D). Overall, our results depicted in Figures 2A–D demonstrate that MPH and *R. officinalis* significantly improved cognition and alleviate the cognitive deficits induced by AlCl_3_.

### MPH and *R. officinalis* Induced Antidepressant Effects

**FIGURE 3:**
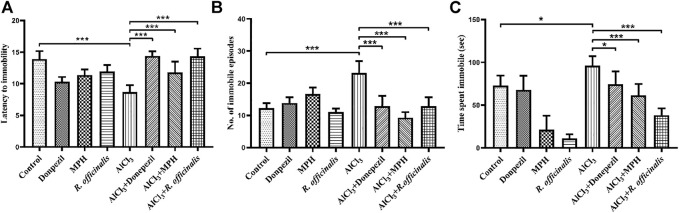
Effects of Donepezil, MPH, and *R. officinalis* on depression-like behavior in the forced swim test. **(A)** Graph depicting latency to immobility, **(B)** number of immobile episodes, and **(C)** time spent immobile. One-way ANOVA followed by the Bonferroni comparison test (mean ± SEM) was applied to analyze the data using GraphPad Prism. n = 8. ^***^ *p* < 0.001, ^*^ *p* < 0.05, ns nonsignificant.

The forced swim test was conducted to assess the antidepressant effect of MPH and *R. officinalis* on an AlCl_3_-induced AD mice model having impaired cognitive functions. The AlCl_3_-treated group exhibited depressive behavior by showing the least latency to immobility as compared with the control and other experimental groups. The AlCl_3_ + MPH-treated group spent significantly more time struggling and hence delayed latency to immobility in comparison with the AlCl_3_-treated group demonstrating that treatment aided in overcoming the effect of AlCl_3_ on depression. However, AlCl_3_ + Donepezil and AlCl_3_ + *R. officinalis* comparatively delayed more (*p* < 0.001) latency to immobility than AlCl_3_ + MPH (Figure 3A). Likewise, the AlCl_3_-treated group displayed the highest number of immobile episodes as compared with all other experimental groups, and after treatment, a significant decrease in the number of immobile episodes was observed in the AlCl_3_ + Donepezil- and AlCl_3_ + *R. officinalis*-treated groups with AlCl_3_ + MPH having the least number of immobile episodes as compared with the other treatment groups (Figure 3B). The AlCl_3_-treated group also spent the greatest amount of time being immobile, which was significantly decreased via drug treatment in the AlCl_3_ + Donepezil- and AlCl_3_ + MPH-treated groups, respectively. A more significant decrease (*p* < 0.05) in time spent being immobile was observed after treatment with *R. officinalis* (Figure 3C). The overall findings depicted in Figures 3A–C show that MPH and *R. officinalis* significantly induce antidepressant effects and reduce depression-like behavior induced by AlCl_3_.

### *R. officinalis* Induced Anxiolytic Effect

**FIGURE 4:**
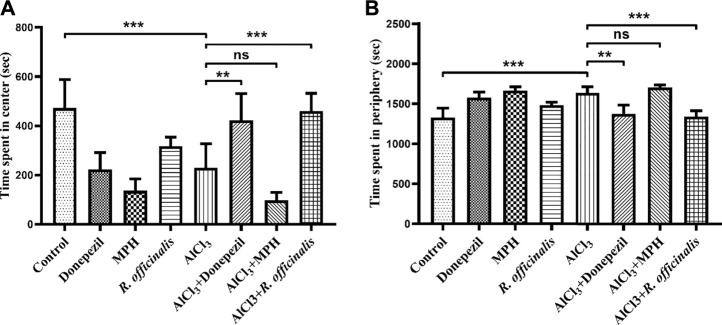
Effects of Donepezil, MPH, and *R. officinalis* on anxiety and exploratory behavior in the open field test. **(A)** Graph depicting time spent in the center and **(B)** time spent in the periphery of the open field box. One-way ANOVA followed by the Bonferroni comparison test (mean ± SEM) was applied to analyze the data using GraphPad Prism. n = 8. ^***^ *p* < 0.001, ^**^ *p* < 0.01.

An open field test was conducted to analyze the anxiety-like behavior in animal models. Animals that tend to spend more time in the center are considered less anxious. The AlCl_3_-treated group showed more anxiety-like behavior by spending significantly (*p* < 0.001) lesser time in the center and more time in the periphery as compared with the control group (Figures 4A, B). The AlCl_3_ + Donepezil- and AlCl_3_ + *R. officinalis*-treated groups improved their performance by spending more time in the center as compared with the AlCl_3_-treated group, demonstrating the antianxiety potential of Donepezil and *R. officinalis*, whereas the AlCl_3_ + MPH-treated group spent the least amount of time in the center of the box (Figure 4A). Likewise, treatment with Donepezil and *R. officinalis* significantly decreased the time spent in the periphery in the AlCl_3_ + Donepezil- and AlCl_3_ + *R. officinalis*-treated groups, respectively, in comparison with the AlCl_3_-treated group (Figure 4B). However, the AlCl_3_ + *R. officinalis*-treated group showed more significant improvement than the AlCl_3_ + Donepezil-treated group. It is of interest that the most anxious behavior was observed after treatment with MPH (Figures 4A, B). These results indicate the potential of *R. officinalis* to induce an anxiolytic effect.

### Effects of MPH and *R. officinalis* on Amyloid Beta Plaque Burden

**FIGURE 5:**
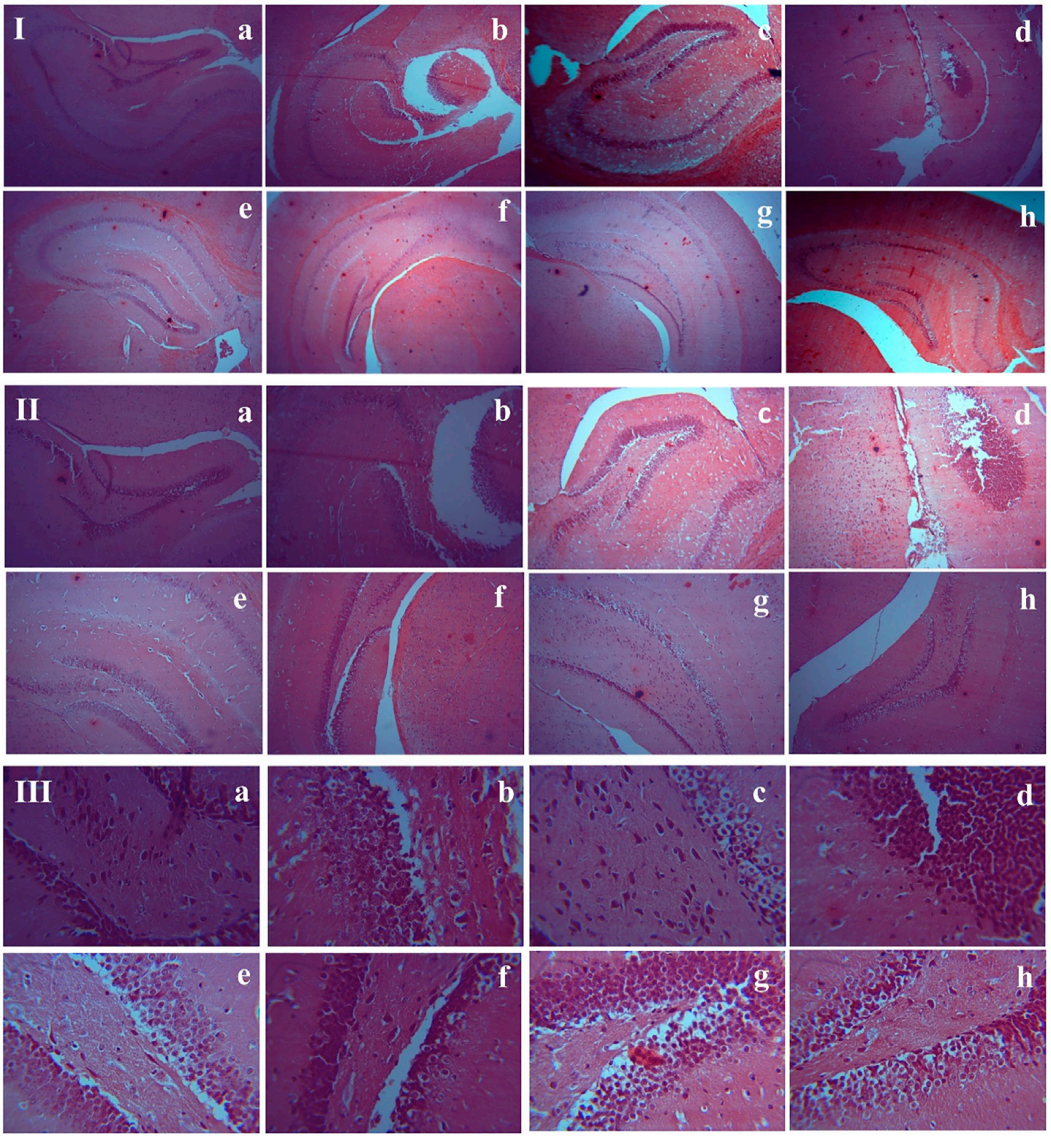
Histological assessment of hippocampal tissues sections stained with Congo Red. **(I)** 4×, **(II)** 10×, **(III)** 40×. **(a)** Control, **(b)** Donepezil-treated, **(c)** MPH-treated, **(d)** *R. officinalis*-treated, **(e)** AlCl_3_-treated, **(f)** AlCl_3_ + Donepezil-treated, **(g)** AlCl_3_ + MPH-treated **(h)** AlCl_3_ + *R. officinalis*-treated.

Congo red staining of the hippocampus showed the presence of Aβ plaques in the AlCl_3_-treated groups compared with the control group. An almost similar number of plaques are seen in all AlCl_3_ plus drug-treated groups and the AlCl_3_-treated group. None of the tested groups showed a significant decrease in Aβ plaque burden (Figure 5). Therefore, it is likely that the selected doses of MPH and *R. officinalis* are not much effective to reduce the amyloid burden.

## Discussion

The current study investigated the effects of MPH and *R. officinalis* on various parameters including memory and learning, anxiety, and depression in the AlCl_3_-induced AD mouse model.

Aluminum (Al) is the most abundant element in earth’s crust and is in human use for centuries. Its widespread use and exposure originate from many sources including vaccine adjuvants, processed foods, cosmetics, medical treatments, cooking wares, and pharmaceuticals (Tietz et al., 2019). Chronic exposure to Al develops cellular toxicity and accumulation in various organs including the central nervous system. It binds to plasma transferrin and citrate molecules in the body and is transferred to the brain. The Al accumulation in the brain generates misfolded proteins and facilitates hyperphosphorylation and aggregation (Colomina and Peris-Samperdo, 2017; Niu 2018). AlCl_3_ is commonly used to develop aluminum-induced animal models for AD, where it is suggested to cause shrinking of hippocampus pyramidal cells (Saeed et al., 2021), neural cell death, and cognitive deficits similar to AD (Zhang 2018).

MPH and other psychostimulants are often used as a therapy to enhance cognitive functions, reduce impulsivity, and induce wakefulness (Carlier et al., 2019). The majority of the studies emphasized that MPH improves cognitive efficiency and influences working memory, inhibitory control, and mental flexibility (Bolfer et al., 2017). Our results also showed a significant effect of MPH in improving spatial learning and memory. We compared the effects of MPH and *R. officinalis* with Donepezil on the AlCl_3_-induced AD mouse model and found significant effects of MPH on spatial memory and reference memory in terms of the number of platform crossings and the number of entries in the target quadrant in the MWM test. *R. officinalis* also exhibit significant improvement in spatial memory as compared with the AlCl_3_-treated group and demonstrated more significant effects in reference memory especially in comparison with Donepezil in terms of time spent in the target quadrant.

Depression is one of the most prevalent behavioral symptoms of AD (Yang et al., 2020). It is of interest that Donepezil showed antidepressant effects in the mouse forced swim test and chronic mild stress model in rats (Papp et al., 2016; Fitzgerald et al., 2020). However, MPH has also revealed significant improvement in depression symptoms in patients with Asperger syndrome (Golubchik et al., 2017). Our study compared the effects of *R. officinalis* with both Donepezil and MPH on depression-like behavior in the AlCl_3_-induced AD model. Comparison of our forced swim results showed that all the tested compounds significantly improved depression-like symptoms in the AlCl_3_-induced AD model; however, *R. officinalis* exhibited significantly more antidepressant potential as compared with MPH and Donepezil by delaying more latency to immobility and spending more time being immobile. This can be credited to the antidepressant-like activity of one of the active compounds of *R*. *officinalis*, i.e., carnosic acid. Recent evidence reveals carnosic acid as a substantial modulator of the ADPN-FGF9 pathway via activation of PPAR-γ in adipocytes, a recently established factor in the development of depression (Wang X. Q. et al., 2021). Likewise, rosmarinic acid has shown antidepressant effects mediated through its increased antioxidant response (Wang J. et al., 2021) exhibiting neuroprotective effects via activation of GABA_A_ receptors (Wang C. C. et al., 2021). In addition, Lataliza et al. suggested the substantial involvement of cannabinoid receptors/PPAR-γ signaling pathways in exerting the antidepressant-like effect of rosmarinic acid (Lataliza et al., 2021).

Moreover, it has been demonstrated that 60–90% of AD patients develop neuropsychiatric symptoms including anxiety disorder that could be treated with acetylcholinesterase inhibitors (AChEIs) (Cummings et al., 2016; Botto et al., 2022). AChEIs have varying effects on anxiety. Besides several disease-modifying effects of various new derivatives of AChEIs, they also possess promising effects on anxiety-like behavior that can be helpful for disease management (Giménez-Llort et al., 2017). Therefore, the current study also assessed the comparative anxiolytic potential of MPH, *R. officinalis*, and Donepezil in the AlCl_3_-induced AD model. Our results showed that treatment with Donepezil and *R. officinalis* significantly improved anxiety-like behavior in the AlCl_3_-treated AD model by increasing the time spent in the center arena of the open field box. A comparison of the results showed that *R. officinalis* displayed the strongest antianxiety potential. *R. officinalis* reduces the extent of anxiety; however, a substantial decrease in neural activity was also observed in a study by Choukairi et al., which can be attributed to the potential of its specific active compounds exerting their effects through modulation of certain neurotransmitter receptors (Choukairi et al., 2019). For instance, two major compounds of *R. officinalis*, i.e., rosmarinic acid and ursolic acid, exhibit anxiolytic and antidepressant-like effects observed in animal studies (Colla et al., 2015; Mirza et al., 2021).

Besides anxiolytic and antidepressant effects of *R. officinalis*, various other therapeutic properties were also documented by various research groups. Some of the recent significant findings are highlighted in Table 1, which represent the potential of *R. officinalis* as antinociceptive, anti-inflammatory, antiapoptotic, anticancer etc. (Table 1).

**TABLE 1:** Pharmacological properties of *R. officinalis*.

Subject	Route	Observed pharmacological effects	Diseased condition	References
Human	Oral	Anticholinesterase, antioxidant	Healthy volunteers	Dabaghzadeh et al. (2022)
Human	Oral	Improvement of mental energy and quality of sleep	Poor mental health	Araki et al. (2020)
Human	Oral	Reduced bacterial plaque and gingival bleeding	Gingival bleeding	Valones et al. (2019)
Humans	Oral	Memory enhancement, antianxiety, antidepressant, improved sleep quality	Healthy volunteers	Nematolahi et al. (2018)
Humans	Oral	Antianxiety, antidepressant	Healthy volunteers	Achour et al. (2022)
Humans	Inhalation	Decreased sleepiness and increased alertness	Sleepiness and alertness	Nasiri & Boroomand (2021)
Mice	Oral Gavage	Antidepressant, anti-inflammatory, rebalanced gut microbiota	Chronic restraint stress, hippocampus inflammation	Guo et al. (2018)
Mice	Oral	Memory improvement, synaptic regulation	Aβ_-1-42_-induced AD	Mirza et al. (2021)
Mice	Oral Gavage	Anticancer	Colorectal cancer (xenograft tumor model)	Valdés et al. (2017)
Mice	Topical application	Antibacterial effect, wound healing	Wound with bacterial infection	Khezri et al. (2019)
Mice	Oral	Antidepressant, anxiolytic, modulation of oxytocinergic system in limbic system	Depression induced by tail suspension test (TST), anxiety model of LPS induced neuroinflammation	Sasaki et al. (2021)
Mice	I.P.	Memory enhancement, anti-inflammatory, neurogenic	AlCl_3_-induced neurotoxicity	Khalid et al. (2020)
Mice	Inhalation	Reduction of stress and corticosterone in serum, increased dopamine level in the brain	Stress	Villareal et al. (2017)
Mice	Oral Gavage	Prevention of kidney function	CCl4-induced nephrotoxicity	Hamed et al. (2020)
Rats	I.P.	Pain relieving, anti-inflammatory	Neuropathic pain induced by chronic constriction injury of the sciatic nerve	Ghasemzadeh Rahbardar et al. (2017)
Rats	I.P.	Anti-inflammatory, antioxidant, reduced fibrosis	Postoperative peritoneal adhesion	Roohbakhsh et al. (2020)
Rats	Oral Gavage	Intestinal protection, antioxidant, anti-inflammatory	Ethanol-induced acute intestinal damage	Amaral et al. (2018)
Rats	Oral Gavage	Anti-inflammatory, antioxidant, reduced paw edema	Freund’s adjuvant-induced arthritis	de Almeida Gonçalves et al. (2018)
Rats	Oral	Decreased bone loss, anti-inflammatory, antioxidant	Osteoporosis	Elbahnasawy et al. (2019)
Rats	Oral	Reduced edema, anti-inflammatory, antialgic	Carrageenan-induced paw edema	Borges et al. (2018)
Rats	Stomach tube	Enhancement of cognitive functions, anti- inflammatory, antioxidant	Ibotenic acid induced AD	Rezk et al. (2022)
Rats	Oral	Memory improvement	Lipopolysaccharide (LPS) induced memory deficits	Pusceddu et al. (2022)
Rats	Oral	Antioxidant	Renal toxicity and oxidative stress	El-Demerdash et al. (2021)
Rats	I.P.	Anxiolytic-like activity	Anxiety	Choukairi et al. (2019)
Rats	Oral Gavage	Neuroprotective, antinociceptive, antihyperalgesic	Diabetic neuropathy	Rasoulian et al. (2019)
Rats	Oral	Antidyslipidemic and antiatherogenic activity	Triton and saturated fat-induced (CSF) dyslipidemias	Santos Rodrigues et al. (2020)

Of note, bioactive compounds of *R. officinalis* have limited ability to cross the blood–brain barrier; therefore, different delivery approaches were experimented with to enhance delivery through the blood–brain barrier. Kuo et al. demonstrated apolipoprotein E-modified liposomes conjugated with phosphatidic acid as a carrier for rosmarinic acid and quercetin to infiltrate the BBB to block Aβ_1–42_-induced AD (Kuo et al., 2018). Likewise, attachment of specific ligand, targeted delivery by nanoparticles, and nanoemulsion through intranasal administration were used to rescue neurodegeneration (Fachel et al., 2020; Kuo et al., 2020; Long et al., 2020).

It is of interest that MPH exhibited increased anxiety-like behavior in the open field test in the current study. The effects of MPH on anxiety are still not clear. **S**ome studies have shown that MPH either decreases (Jager et al., 2019) or increases anxiety (Zoratto et al., 2019). Different effects of MPH on anxiety have been debated, and such variations indicate the multidimensional and complex nature of emotional behaviors. There is a possibility that the procedures applied to assess anxiety only measure a specific idiosyncratic domain of the tested emotion. For instance, as reported by Boyette-Davis et al., the antianxiety effects of MPH are not affected based on the changes in locomotor behavior (Boyette-Davis et al., 2018). Therefore, various experimental approaches would be needed to completely assess the effects of MPH on anxiety. By contrast, acute administration of MPH has also been found to exhibit anxiolytic effects in the open field test, where MPH was administered 20 min prior to testing (Jager et al., 2019). Differences in dosage and treatment duration could be another possible reason for varying effects on anxiety (Zoratto et al., 2019). Although the physiological and behavioral effects of MPH are generally reversible after intermittent MPH chronic exposure, it is followed by prolonged abstinence (Kalinowski et al., 2020). However, withdrawal effects could also be the possible reason for high anxiety in the MPH-treated group in the present study.

A histopathological assessment revealed the formation of amyloid plaques in the AlCl_3_-treated groups showing the development of AD pathology. Our results revealed that all the tested compounds failed to reduce the Aβ plaques burden. Although carnosic acid significantly improves cholinergic dysfunction and mitochondrial defects by reducing the Aβ-mediated toxicity (Chen et al., 2022), in our case, no substantial effect was observed by any of the tested drugs or *R. officinalis.* Perhaps the selected doses of our study are not much effective to reduce the Aβ plaques with a shorter treatment duration, which could be the possible reason for not being effective in reducing the amyloid burden.

These findings provide a preliminary data set on the therapeutic potential of *R. officinalis* possessing substantial anxiolytic and antidepressant activities. MPH and *R. officinalis* have significant effects on cognition with MPH being more effective than *R. officinalis* in restoring spatial memory. Nevertheless, MPH increases the anxiety-like behavior, which needs a further understanding of the complex molecular mechanisms involved in its mode of action.

## Conclusion

Memory loss along with neuropsychiatric symptoms in AD leads to the requirement of a drug that can enhance memory and reduce behavioral despair. Donepezil has failed to stop the disease progression and is associated with various side effects, and MPH can aggravate psychological symptoms. Therefore, *R. officinalis*, a natural plant extract, could be more suitable to treat cognitive decline as well as psychological symptoms with minimal side effects. Further investigation of the mode of action of *R. officinalis* and the role of its various active compounds is warranted to indicate its therapeutic potential for AD.

## Data Availability

The original contributions presented in the study are included in the article/Supplementary Material. Further inquiries can be directed to the corresponding author.
